# Bound states in the continuum in anisotropic photonic crystal slabs

**DOI:** 10.1038/s41598-023-40869-0

**Published:** 2023-08-29

**Authors:** Ruey-Lin Chern, Jui-Chien Chang, Hsueh-Chi Yang

**Affiliations:** Institute of Applied Mechanics, National Taiwan University, Taipei, 106 Taiwan

**Keywords:** Photonic crystals, Nanophotonics and plasmonics

## Abstract

We investigate the bound states in the continuum (BICs) in photonic crystal slabs composed of alternating anisotropic and isotropic dielectric materials. According to the orientation of optical axis plane, three different configurations are proposed for analyzing various types of BICs, associated with extremely large quality factors and vanishing spectral linewidths. In particular, symmetry-protected (SP) BICs exist at the Brillouin zone center for zero rotation angle of the optical axis, which exhibit antisymmetric field patterns that are decoupled from the symmetric radiating fields. Accidental BICs and Friedrich-Wintgen (FW) BICs also occur at the Brillouin zone center for particular rotation angles of the optical axis. The former emerge on isolated bands with quasi-symmetric or quasi-antisymmetric field patterns, while the latter appear near the avoided crossing between two dispersion bands. At off the Brillouin zone center, SP BICs do not exist while accidental BICs and FW BICs appear at particular optical axis rotation angles, with similar features but somewhat more asymmetric field patterns than those at the Brillouin zone center.

## Introduction

Bound states in the continuum (BICs) are non-leaky localized resonance modes that coexist with a continuous spectrum of radiating waves^1–4^. Since BICs will not radiate to the far field, they are decoupled from the far-field radiation and possess infinite quality factors with zero spectral linewidths. In the presence of the absorption loss, roughness, or finite size of the structure, a theoretically true BIC is turned into a quasi-BIC with a finite quality factor. In 1929, von Neumann and Wigner discovered that one-dimensional (1D) potential can support localized solutions that correspond to isolated discrete eigenvalues embedded in the continuum of positive energy states^5^. In 1985, Friedrich and Wintgen proposed the concept of BIC as a result of complete destructive interference of two resonances undergoing an avoided crossing. When two resonant states approach each other as a function of certain continuous parameter, the resonance width of one of them vanishes and forms the so-called Friedrich-Wintgen (FW) BIC^6^. In recent years, BICs have been found in quantum^7,8^, photonic^9–12^, acoustic and water wave^13,14^, and mathematical^15^ systems, which can be applied in a variety of areas^4^, including integrated photonic circuits^16^, filters^17^, lasers^18^, and biosensors^19^.

BICs can be generally categorized into two types: symmetry-protected (SP) BICs and accidental BICs. The SP BICs appear at the center of Brillouin zone for a periodic lattice usually with perfect symmetry in geometry^20,21^. The accidental BICs are found at certain wave vector points on isolated dispersion bands when the relevant coupling to the radiation continuum completely vanishes^22^. The accidental BICs can be further divided into two major groups: Fabry-Perot BICs and FW BICs^23^. The former are formed by two interacting objects at one resonance while the latter are formed by two interacting resonances at one object^1^. In particular, FW BICs are generally found in the vicinity of the avoided crossing of two dispersion bands, arise because of the destructive interference of two resonances coupled to the same radiation channel^24^. Single-resonance parametric BICs^1^ and resonance-trapped BICs^25^ are also considered as accidental BICs.

Different types of BICs originate from distinct physical mechanisms and occur in a variety of photonic systems, including gratings^26–28^, waveguides^29^, metasurfaces^30–32^, photonic crystals^33,34^, and photonic crystal slabs^22,35–39^. In particular, 1D photonic crystal slabs^10,25,40–44^ are considered one of the most simple structures that support various types of BICs, which have been theoretically identified and experimentally observed^25,29,41,42^. The formation mechanism of BICs in the photonic crystal slabs has been interpreted by the destructive interference of radiation from the bulk states in the slab^25,29,41,43^. The BICs are the eigenmodes lying above the light cone, despite that they are completely guided without radiative leakage^25^. The absence of leakage originates from two different physical mechanisms: symmetry incompatibility for the SP BICs and destructive interference between different leakage channels for the accidental BICs^22^.

In contrast to isotropic structures with a single radiation channel, the resonance modes in anisotropic structures exhibit two possible radiation channels^45^, which correspond to the ordinary and extraordinary waves. The resonance modes are usually leaky in the continuous frequency spectrum^1^. As BICs may occur when the radiation channel of leaky mode is suppressed, the anisotropy in the structure provides additional parameters to be tuned for destructive interference of the radiative waves to occur^45,46^. The ordinary and extraordinary refractive indices in the anisotropic material may alter the strength of electric field components. The orientation of optical axis can further rotate the field components^47–51^. As a consequence, the anisotropic structure acquire more system parameters in the tuning process to form the BICs.

In the present study, we investigate the BICs in photonic crystal slabs that compose of alternating anisotropic and isotropic dielectric materials. To analyze various types of BICs in the underlying structure, three configurations according to the orientation of optical axis plane are proposed. Basic features of BICs as extremely large quality factors and vanishing spectral linewidths are examined in the dispersion bands and transmittance diagram. At the Brillouin zone center ($k_x=0$), SP BICs exist for zero rotation angle of the optical axis, which exhibit antisymmetric field patterns that are decoupled from the symmetric radiating fields. Accidental BICs and FW BICs occur at particular rotation angles of the optical axis. The former emerge at certain wave vector points on isolated bands with quasi-symmetric or quasi-antisymmetric field patterns, while the latter appear near the avoided crossing between two dispersion bands. At off the Brillouin zone center ($k_x\ne 0$), SP BICs do not exist while accidental BICs and FW BICs appear at particular optical axis rotation angles. Compared to BICs at $k_x=0$, BICs at $k_x\ne 0$ possess similar features but somewhat more asymmetric field patterns.

**Figure 1:**
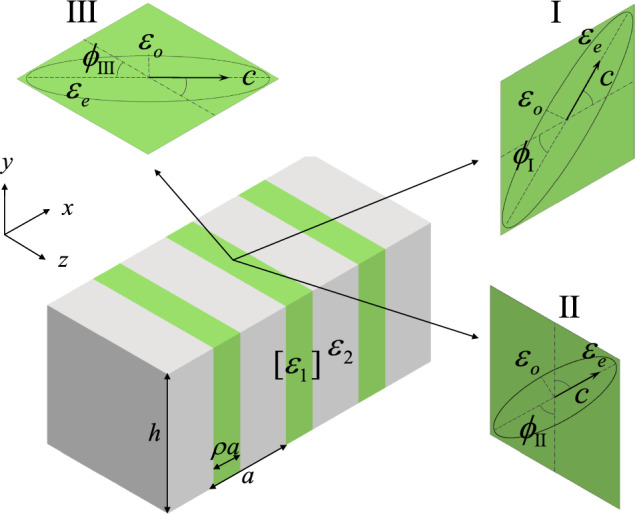
Schematic diagram of the anisotropic photonic crystal slab, where the anisotropic and isotropic materials are shaded in green and gray colors, respectively. Insets are ellipse representations of the uniaxially anisotropic material for three different configurations.

## Results

**Anisotropic photonic crystal slabs.** Consider a photonic crystal slab composed of alternating anisotropic and isotropic dielectric materials, as schematically shown in Fig. 1, where *a* is the lattice constant, *h* is the slab height, $[\varepsilon _1]$ is the dielectric tensor of the anisotropic material with the portion $\rho$ in the unit cell, and $\varepsilon _2$ is the dielectric constant of the isotropic material. Assume that the anisotropic material is uniaxial, with $\varepsilon _o$ and $\varepsilon _e$ being the ordinary and extraordinary dielectric constants, respectively. According to the orientation of optical axis plane, there exist three different configurations for the anisotropic material. Here, I, II, III refer to the configurations where the optical axis vector $\textbf{c}$ lies on the *xy*, *yz*, *xz* planes, with $\phi _{\textrm{I}}$, $\phi _{\textrm{II}}$, $\phi _{\textrm{III}}$ being the rotation angles of the vector $\textbf{c}$ with respect to the *x*, *y*, *z* axes, respectively. As the photonic crystal slabs are connected structures similar to the dielectric gratings, no substrate is present^25,43^ in order to give more concise resonance features associated with BICs. In the presence of a substrate, the basic features of BICs will retain except that they are red-shifted and the associated resonances are slightly less significant.

To identify various types of the BICs, we compute the eigenfrequencies by the free and open-source software package MPB (MIT Photonic Bands) based on the plane wave expansion method and the supercell approach^52^, which have been extensively used in the study of photonic crystal slabs. To analyze the features of BICs, we also calculate the transmittance (ratio of the transmitted to incident power) based on the finite element method, which has been employed to study the transmission characteristics of grating structures and photonic crystal slabs^53–55^. The Bloch boundary condition is applied at the unit cell boundary and perfectly matched layers are applied on top and bottom of the computational domain^56^. An important factor for evaluating BICs is the quality factor (*Q* factor) based on the eigenfrequency $\Lambda$, which is defined as $Q=\textrm{Re}[\Lambda ]/\left( 2\textrm{Im}[\Lambda ]\right)$^57^, where $\textrm{Re}[\cdot ]$ and $\textrm{Im}[\cdot ]$ denote the real and imaginary parts, respectively. Another definition of the quality factor is based on the spectral response as $Q=f_r/\Delta f_r$, where $f_r$ is the resonance frequency and $\Delta f_r$ is the linewidth (full width at half maximum) of the transmittance (or reflectance). The above two definitions of the quality factor are nearly equivalent and amount to the ratio of energy stored in the resonator to the energy dissipated per cycle by damping processes^58^. A larger quality factor corresponds to a narrower linewidth with a smaller damping rate and a longer lifetime for the resonator. The higher the quality factor, the stronger the interaction between the confined light field and the material or structure. Theoretically, the linewidth of an ideal BIC becomes zero and the quality factor tends to infinity^22,59^.

In the present study, we report the results of BICs in the anisotropic photonic crystal slabs for transverse magnetic (TM) polarization. Here, TM refers to the case where the magnetic field is perpendicular to the longitudinal plane formed by the crystal lattice and the slab height, that is, the *xy* plane. The BICs in isotropic photonic crystal slabs for TE polarization, where the electric field is perpendicular to the longitudinal plane, can be found in Ref.^25^.

**Figure 2:**
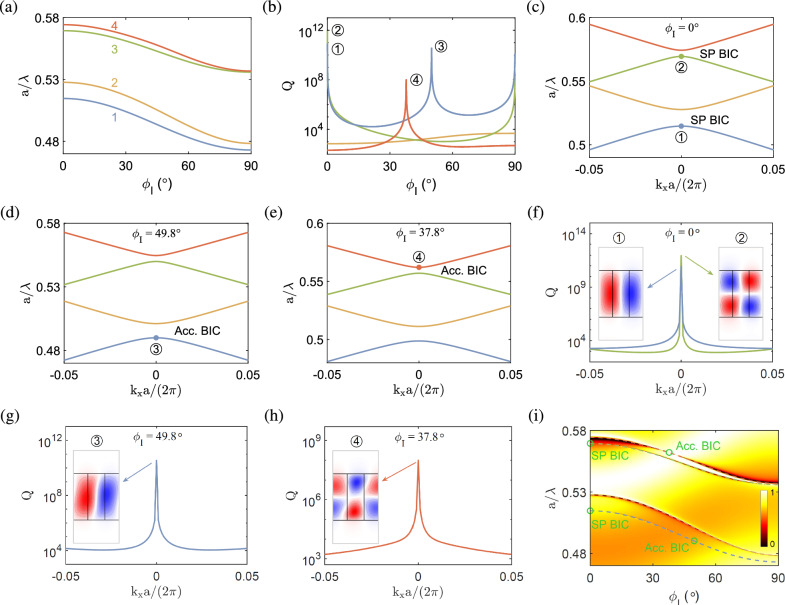
BICs in the anisotropic photonic crystal slab at $k_x=0$ in configuration I, where $\varepsilon _o=5.2$, $\varepsilon _{e}=8.2$, $\varepsilon _2=3.3$, $\rho =0.37$, and $h/a=1.66$. (**a**) Normalized frequencies and (**b**) quality factors as functions of the rotation angle $\phi _{\mathrm{{I}}}$. (**c**)–(**e**) Band structures for $\phi _{\mathrm{{I}}}=0^\circ$, $49.8^\circ$, $37.8^\circ$, respectively. (**f**)–(**h**) Quality factors as functions of the wave number and transverse magnetic fields ($\textrm{Re}[H_z]$) of the SP BICs in (**c**) and the accidental BICs in (**d**), (**e**), respectively. Blue and red colors represent positive and negative values, respectively. (**i**) Transmittance diagram as a function of the normalized frequency and rotation angle.

**1. BICs at the Brillouin zone center** ($k_x=0$)

**Configuration I.** In this configuration, the optical axis vector lies on the *xy* plane (cf. Fig. 1) and the permittivity tensor of the anisotropic medium in Cartesian coordinate system is given by^60^

1 $$\begin{aligned} \begin{array}{l} [{\varepsilon _1}] = [R_{\textrm{I}}][{\varepsilon _{\textrm{I}}}]{[R_{\textrm I}]^T} \end{array}, \end{aligned}$$

where [$$\varepsilon _{\textrm{I}}]=\textrm{diag}[\varepsilon _e,\varepsilon _o,\varepsilon _o]$$ and

2 $$\begin{aligned} {R_{\textrm{I}}} = \left[ {\begin{array}{*{20}{c}} {\cos {\phi _{\textrm{I}}}} &{} { - \sin {\phi _{\textrm{I}}}} &{} 0 \\ {\sin {\phi _{\textrm{I}}}} &{} {\cos {\phi _{\textrm{I}}}} &{} 0 \\ 0 &{} 0 &{} 1 \\ \end{array}} \right] \end{aligned}$$

is the transformation matrix used to perform a rotation of angle $\phi _{\textrm{I}}$ about the *z* axis^61^. Here, $\textrm{diag}[\cdot ,\cdot ,\cdot ]$ denotes a 3 $\times$ 3 diagonal matrix.

Figure 2a shows the normalized frequencies ($a/\lambda$) of four relevant dispersion bands for the anisotropic photonic crystal slab in configuration I at the Brillouin zone center ($k_x=0$) as functions of the rotation angle $\phi _{\textrm{I}}$. The frequencies are lowered as the rotation angle increases. In Fig. 2b, extremely large quality factors are attained at their peaks on band 1 (blue line) and band 3 (green line) for zero rotation angle ($\phi _{\textrm{I}}=0^\circ$), which are labeled by $\textcircled {1}$ and $\textcircled {2}$, respectively. Because of symmetry in the ellipse representation of the anisotropic medium, the quality factor curves are symmetric about $\phi _{\textrm{I}}=90^\circ$ and therefore two similar peaks will occur at $\phi _{\textrm{I}}=180^\circ$. In addition, the quality factor curves in the range of $\phi _{\textrm I}=0^\circ$ to $180^\circ$ coincide with those in the range of $\phi _{\textrm{I}}=180^\circ$ to $360^\circ$.

The band structure near the Brillouin zone center for $\phi _{\textrm I}=0^\circ$ is shown in Fig. 2c. The transverse magnetic fields ($\textrm{Re}[H_z]$) of the eigenmodes at $\textcircled {1}$ and $\textcircled {2}$, as plot in Fig. 2f, display *antisymmetric* (TM$_{21}$-like and TM$_{22}$-like) patterns, which is characteristic of the SP BICs^25,62,63^. Here, the pattern symmetry is viewed along the periodic direction (*x* axis)^25^, and the magnetic fields are plot in one unit cell that consists of the anisotropic medium of width $\rho a$ in the middle and the isotropic medium of width $\left( 1-\rho \right) a/2$ on the left and right sides. The TM$_{mn}$-like refers to the field pattern (in the unit cell) similar to the mode structure (in the cross section) in rectangular waveguides, where *m* and *n* are mode numbers in the horizontal and vertical directions, respectively^58^. The transverse magnetic fields of the BICs are well confined in the slab because of the dielectric contrast between inside and outside the slab, which are similar to those in metallic waveguides.

The SP BICs originates from the symmetry incompatibility and necessarily have an antisymmetric field pattern [$H_z(x,y)=-H_z(-x,y)$], so that they will not couple to the symmetric radiating fields. For this reason, the SP BICs always occur at $k_x = 0$ and cannot exist at $k_x \ne 0$^25^. Note that the SP BICs also occur at $\phi _{\textrm I}=90^\circ$ [cf. blue and green lines in Fig. 2b] with very similar quality factors and transverse magnetic field patterns, where the optical axis vector is perpendicular to that for $\phi _{\textrm{I}}=0^\circ$. This feature has also been observed in semi-infinite 1D photonic crystals with anisotropic defect layers^48^.

The BICs at $k_x=0$ correspond to the bound states that occur at normal incidence. To evaluate the BICs from the aspect of electromagnetic radiation, the transmittance as a function of the frequency and rotation angle $\phi _{\textrm{I}}$ is plot in Fig. 2i. The dispersion bands [cf. Fig. 2a] are overlaid in the same diagram to show consistent trends with the transmittance. Theoretically, the SP BICs are ideal bound states with infinite quality factors and zero spectral linewidths^22,59^. It is therefore hard to identify such BICs merely from the transmittance diagram without the help of dispersion bands. In fact, the ideal SP BICs cannot be excited from the far field radiation, as the former are decoupled from the latter. As a result, the corresponding transmittance is equal to unity, for no interactions occur between the incident field and the structure. In practice, a certain asymmetry either in the structure or incident angle allows for a small coupling between the bound state and incident field, which turns the ideal BICs into quasi-BICs. The transmittance will be somewhat less than unity and usually featured with the Fano resonance^49^. In this situation, the quality factor of a quasi-BIC is finite (though very large) and its spectral linewidth is not zero (though very small). This is also the circumstance when the quasi-BICs are used for practical applications, for instance, in sensors or filters.

**Figure 3:**
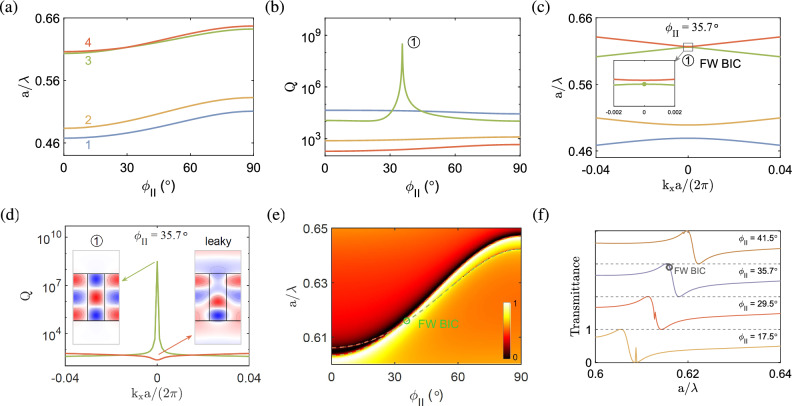
BICs in the anisotropic photonic crystal slab at ${k_x=0}$ in configuration II, with the same geometry and material parameters as in Fig. 2. (**a**) Normalized frequencies and (**b**) quality factors as functions of the rotation angle $\phi _{\mathrm{{II}}}$. (**c**) Band structure for $\phi _{\mathrm{{II}}}=35.7^\circ$. Inset is an enlarged view of two upper bands near ${k_x=0}$, with the green dot corresponding to the FW BIC. (**d**) Quality factor as a function of the wave number and transverse magnetic field ($\textrm{Re}[H_z]$) of the FW BIC in (**c**). (**e**) Transmittance diagram as a function of the normalized frequency and rotation angle. (**f**) Transmittance curves as functions of the normalized frequency for selected rotation angles.

In Fig. 2b, very large quality factors are attained at their peaks on band 1 (blue line) and band 4 (orange line) for particular rotation angles ($\phi _{\textrm{I}}=49.8^\circ$ and $37.8^\circ$), which are labeled by $\textcircled {3}$ and $\textcircled {4}$, respectively. The corresponding band structures near the Brillouin zone center are shown in Fig. 2d and e. Note that the eigenmodes with quality factor peaks occur on *isolated* bands^64^, which is characteristic of the accidental BICs^25,40,41^. This type of BICs are also named single-resonance parametric BICs^1,22^, which usually occur at oblique incidence^65^ and are often referred to as off-$\Gamma$ BICs^64^. It is, however, possible to create the accidental BICs at ${k_x=0}$ (or under normal incidence) because of the interference between two or more modes, which have been observed in 1D photonic crystal slabs^25^. In the present study, the accidental BICs also occur at ${k_x=0}$ for particular rotation angles of the anisotropic material. The transverse magnetic fields ($\textrm{Re}[H_z]$) of the eigenmodes at $\textcircled {3}$ and $\textcircled {4}$ are plot in Fig. 2g and h, respectively, which display a quasi-antisymmetric (TM$_{21}$-like) or quasi-symmetric (TM$_{32}$-like) pattern. Because of the material anisotropy, no strict symmetry is presented in the field distribution, although the symmetric or antisymmetric pattern is almost, but not exactly, fulfilled. In photonic crystal slabs, the accidental BICs originate from the interference between two or more Bloch waves bouncing back and forth vertically inside the slab^25^. It can be examined in Fig. 2i that the accidental BICs correspond to the transmittance peaks with vanishing linewidths^29^. This feature is consistent with the fact that most electromagnetic fields of the BICs are confined in the structure [cf. Fig. 2f–h] and will not radiate to the far field. Equivalently, the BICs are decoupled from the far field radiation.

**Configuration II.** In this configuration, the optical axis vector lies on the *yz* plane (cf. Fig. 1) and the permittivity tensor of the anisotropic medium in Cartesian coordinate system is given by^60^

3 $$\begin{aligned} \begin{array}{l} [{\varepsilon _1}] = [R_{\textrm{II}}][{\varepsilon _{\textrm{II}}}]{[R_{\textrm II}]^T} \end{array}, \end{aligned}$$

where $$[{\varepsilon _{\textrm{II}}}]=\textrm{diag}[\varepsilon _o,\varepsilon _e,\varepsilon _o]$$ and

4 $$\begin{aligned} {R_{\textrm{II}}} = \left[ {\begin{array}{*{20}{c}} 1 &{} 0 &{} 0 \\ 0 &{} {\cos {\phi _{\textrm{II}}}} &{} { - \sin {\phi _{\textrm{II}}}} \\ 0 &{} {\sin {\phi _{\textrm{II}}}} &{} {\cos {\phi _{\textrm{II}}}} \\ \end{array}} \right] \end{aligned}$$

is the transformation matrix used to perform a rotation of angle $\phi _{\textrm{II}}$ about the *x* axis^61^. This configuration has also been used in the study of BICs in semi-infinite 1D photonic crystals with anisotropic defect layers^48,49^.

Figure 3a shows the normalized frequencies ($a/\lambda$) of four relevant dispersion bands for the anisotropic photonic crystal slab in configuration II at the Brillouin zone center ($k_x=0$) as functions of the rotation angle $\phi _{\textrm{II}}$. The frequencies are raised as the rotation angle increases. In Fig. 3b, a very large quality factor is attained at its peak on band 3 (green line) for a particular rotation angle ($\phi _{\textrm II}=35.7^\circ$), which is labeled by $\textcircled {1}$. The corresponding band structure near the Brillouin zone center is shown in Fig. 3c. Note that the *avoided crossing* occurs between band 3 (green line) and band 4 (orange line) (with an enlarged view in the inset), which is characteristic of the FW BICs^29,63^. In particular, the quality factor for one of the two bands goes to a very large value near the avoided crossing point, while the quality factor for the other band is much smaller [cf. Fig. 3b]. The transverse magnetic fields ($\textrm{Re}[H_z]$) of the eigenmodes near $\textcircled {1}$ with the quality factor peak, as plot in Fig. 3d, display a bound state on one band (green line) and a leaky state on the other band (orange line)^63^. The former is well localized in the slab with a symmetric (TM$_{33}$-like) pattern, while the latter is radiative outside the slab.

**Figure 4:**
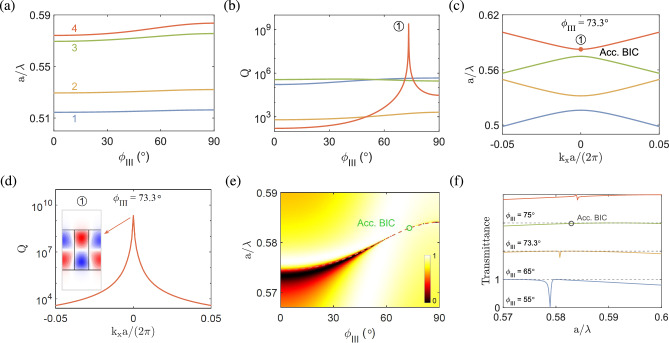
BICs in the anisotropic photonic crystal slab at ${k_x=0}$ in configuration III, with the same geometry and material parameters as in Fig. 2. (**a**) Normalized frequencies and (**b**) quality factors as functions of the rotation angle $\phi _{\mathrm{{III}}}$. (**c**) Band structure for $\phi _{\mathrm{{III}}}=73.3^\circ$. (**d**) Quality factor as a function of the wave number and transverse magnetic field ($\textrm{Re}[H_z]$) of the accidental BIC in (**c**). (**e**) Transmittance diagram as a function of the normalized frequency and rotation angle. (**f**) Transmittance curves as functions of the normalized frequency for selected rotation angles.

Unlike the accidental BICs that are attributed to single resonances, the FW BICs are formed by tuning the interaction between two or more resonances^39^. This property can be understood from the coupled mode theory for a simple system with two resonances: $i{\partial A}/{\partial t}={{\mathcal {H}}}A$^1^, where $A=\left( A_1,A_2\right) ^T$ are the amplitudes of resonances and

5 $$\begin{aligned} H = \left( {\begin{array}{*{20}{c}} {{\omega _1}} &{} \kappa \\ \kappa &{} {{\omega _2}} \\ \end{array}} \right) - i\left( \begin{array}{*{20}{c}} {{\gamma _1}} &{} {\sqrt{{\gamma _1}{\gamma _2}} } \\ {\sqrt{{\gamma _1}{\gamma _2}} } &{} {{\gamma _2}} \\ \end{array} \right) \end{aligned}$$

is the Hamiltonian of the system. Here, $\omega _i$ and $\gamma _i$ ($i = 1, 2$) are the resonant frequencies and damping rates, respectively, of the *i*-th resonance, and $\kappa$ is the coupling strength between the two resonances. If the following condition is satisfied^1^:

6 $$\begin{aligned} \kappa ({\gamma _1} - {\gamma _2}) = \sqrt{{\gamma _1}{\gamma _2}} ({\omega _1} - {\omega _2}), \end{aligned}$$

the eigensystem for the Hamiltonian *H* is solved to give a real eigenvalue ${\omega _ r } = \left( {{ {\gamma _1}{\omega _2}- {\gamma _2}{\omega _1} }}\right) /\left( {{{\gamma _1} - {\gamma _2}}}\right)$ and a complex eigenvalue ${\omega _c } = \left( {{{\gamma _1}{\omega _1} - {\gamma _2}{\omega _2}}}\right) /\left( {{{\gamma _1} - {\gamma _2}}}\right) - i\left( {{\gamma _1} + {\gamma _2}} \right)$. The eigenvalue with a purely real part turns into a BIC and the other eigenvalue, with the imaginary part equal to the sum of two damping rates, becomes more lossy. This is the typical feature of FW BICs^6^. Note that the above solutions for the FW BICs are valid when ${\omega _1} \approx {\omega _2}$ and ${\gamma _1} \approx {\gamma _2}$ [cf. Eq. (6)], that is, the frequencies and damping rates of the two resonances are roughly equal.

Figure 3e shows the transmittance diagram as a function of the normalized frequency and rotation angle $\phi _{\textrm{II}}$. The dispersion bands [cf. Fig. 3a] are overlaid in the same diagram to show consistent trends with the transmittance. The feature of avoided crossing can be observed as two resonances approach each other at a particular rotation angle. To illustrate the FW BIC in a more clear manner, the transmittance curves as functions of the normalized frequency for selected rotation angles are plot in Fig. 3f. Note that the FW BIC [corresponding to the peak of quality factor in Fig. 3b] occurs near the transmittance peak, which is accompanied with a transmittance dip to form the feature of Fano resonance. The FW BIC can be located around or even right at the transmittance peak^24,39,63,64^. As the destructive interference in the FW BIC is hardly perfect in real structures, the corresponding transmittance may be slightly less than (though very close to) unity and the FW BIC has a finite yet very large quality factor. In isotropic photonic crystal slabs, the FW BICs occur at $k_x\ne 0$^25^. In the present study, however, the FW BICs are observed at $k_x=0$, as a consequence of additional degree of freedom in the system introduced by the anisotropic material parameters.

**Configuration III.** In this configuration, the optical axis vector lies on the *xz* plane (cf. Fig. 1) and the permittivity tensor of the anisotropic medium in Cartesian coordinate system is given by^60^

7 $$\begin{aligned} \begin{array}{l} [{\varepsilon _1}] = [R_{\textrm{III}}][{\varepsilon _{\textrm{III}}}]{[R_{\textrm III}]^T} \end{array}, \end{aligned}$$

where $$[{\varepsilon _{\textrm{III}}}]=\textrm{diag}[\varepsilon _o,\varepsilon _o,\varepsilon _e]$$ and

8 $$\begin{aligned} {R_{\textrm{III}}} = \left[ {\begin{array}{*{20}{c}} {\cos {\phi _{\textrm{III}}}} &{} 0 &{} {\sin {\phi _{\textrm{III}}}} \\ 0 &{} 1 &{} 0 \\ {\mathrm{{ - }}\sin {\phi _{\textrm{III}}}} &{} 0 &{} {\cos {\phi _{\textrm{III}}}} \\ \end{array}} \right] \end{aligned}$$

is the transformation matrix used to perform a rotation of angle $\phi _{\textrm{III}}$ about the *y* axis^61^ (cf. Fig. 1).

**Figure 5:**
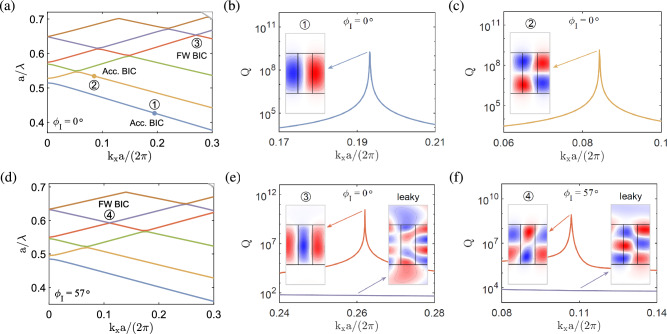
BICs in the anisotropic photonic crystal slab at ${k_x\ne 0}$ in configuration I, with the same geometry and material parameters as in Fig. 2. (**a**) and (**d**) Band structures for ${\phi _{\mathrm{{I}}}=0^{\circ }}$ and $57^{\circ }$, respectively. (**b**), (**c**) and (**e**) Quality factors as functions of the wave number and transverse magnetic fields ($\textrm{Re}[H_z]$) of the accidental BICs at $k_xa/\left( 2\pi \right) =0.195,0.085$ and the FW BIC at $k_xa/\left( 2\pi \right) =0.263$, respectively, for ${\phi _{\mathrm{{I}}}=0^{\circ }}$. (**f**) Quality factor and transverse magnetic field of the FW BIC at $k_xa/\left( 2\pi \right) =0.107$ for $\phi _{\mathrm{{I}}}=57^{\circ }$.

Figure 4a shows the normalized frequencies ($a/\lambda$) of four relevant dispersion bands for the anisotropic photonic crystal slab in configuration III at the Brillouin zone center ($k_x=0$) as functions of the rotation angle $\phi _{\textrm{III}}$. The frequencies do not change much as the rotation angle increases. In Fig. 4b, a very large quality factor is attained at its peak on band 4 (orange line) for a particular angle ($\phi _{\textrm III}=73.3^\circ$), which is labeled by $\textcircled {1}$. The corresponding band structure near the Brillouin zone center is shown in Fig. 4c. Note that the eigenmode with the quality factor peak occurs on an isolated band, which is characteristic of the accidental BICs similar to those in configuration I [cf. Figs. 2d and e]. The transverse magnetic field ($\textrm{Re}[H_z]$) of the eigenmode at $\textcircled {1}$, as plot in Fig. 4d, displays a quasi-symmetric (TM$_{32}$-like) pattern, which is similar to that configuration I [cf. in Fig. 2h].

The transmittance diagram as a function of the normalized frequency and rotation angle $\phi _{\textrm{III}}$ is plot in Fig. 4e. The dispersion bands [cf. Fig. 4a] are overlaid in the same diagram to show consistent trends with the transmittance. To illustrate the accidental BIC in a more clear manner, the transmittance curves as functions of normalized frequency for selected rotation angles are plot in Fig. 4f. Note that the accidental BIC, which corresponds to the peak of quality factor in Fig. 4b, emerges at a frequency where the transmittance curve becomes flat and varies smoothly across the resonance without a noticeable change. The accidental BIC is associated with a high transmittance nearly equal to unity^29,40^, as a consequence of destructive interference in the formation of a BIC.

**Figure 6:**
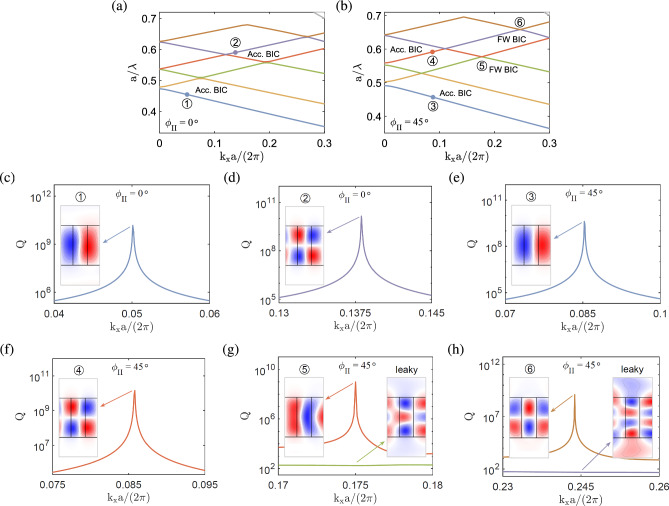
BICs in the anisotropic photonic crystal slab at ${k_x\ne 0}$ in configuration II, with the same geometry and material parameters as in Fig. 2. (**a**, **b**) Band structures for ${\phi _{\mathrm{{II}}}=0^{\circ }}$, $45^{\circ }$. (**c**–**f**) Quality factors and transverse magnetic fields ($\textrm{Re}[H_z]$) of the accidental BICs at $k_xa/\left( 2\pi \right) =0.0501, 0.138$ for ${\phi _{\mathrm{{II}}}=0^{\circ }}$ and $k_xa/\left( 2\pi \right) =0.088, 0.087$ for ${\phi _{\mathrm{{II}}}=45^{\circ }}$, respectively. (**g**, **h**) Quality factors and transverse magnetic fields of the FW BICs at $k_xa/\left( 2\pi \right) =0.175, 0.244$ for ${\phi _{\mathrm{{II}}}=45^{\circ }}$.

**2. BICs off the Brillouin zone center **($k_x\ne 0$)

**Configuration I.** Figure 5a shows the band structure of the anisotropic photonic crystal slab in configuration I for zero rotation angle ($\phi _{\textrm{I}}=0^\circ$). In Fig. 5b and c, very large quality factors are attained at their peaks on two isolated bands (blue and yellow lines) at particular wave numbers [$k_xa/\left( 2\pi \right) =0.195,0.085$], which are labeled by $\textcircled {1}$ and $\textcircled {2}$, respectively. At $k_x \ne 0$, the resonance modes do not present strict symmetry, even though they look approximately symmetric or antisymmetric, which will be in general coupled to the radiation in a continuous frequency spectrum. However, the radiative leakage may be exactly suppressed at certain wave vector components and disappears ’accidentally’ because of the destructive interference between different channels, leading to the accident BICs^22^. These BICs are basically similar to those in Fig. 2, except that the former occur at a particular $k_x$ for $\phi _{\textrm{I}}=0^\circ$, while the latter occur at $k_x=0$ for a particular $\phi _{\textrm{I}}$. The transverse magnetic fields ($\textrm{Re}[H_z]$) of the eigenmodes at $\textcircled {1}$ and $\textcircled {2}$ display quasi-antisymmetric (TM$_{21}$-like and TM$_{22}$-like) patterns [cf. insets in Fig. 5b and c]. On the other hand, the avoided crossing occurs between two upper bands (orange and purple lines) at a particular wave number [$k_xa/\left( 2\pi \right) =0.263$], which is labeled by $\textcircled {3}$. In Fig. 5e, a very large quality factor is attained at its peak on one of the two bands (orange line) that form the avoided crossing, which is characteristic of the FW BIC similar to the case for $k_x=0$ [cf. Fig. 3]. The transverse magnetic fields ($\textrm{Re}[H_z]$) of the eigenmodes near $\textcircled {3}$ display a bound state with a quasi-antisymmetric (TM$_{21}$-like) pattern and a leaky state that largely loses the symmetry [cf. insets in Fig. 5e].

**Figure 7:**
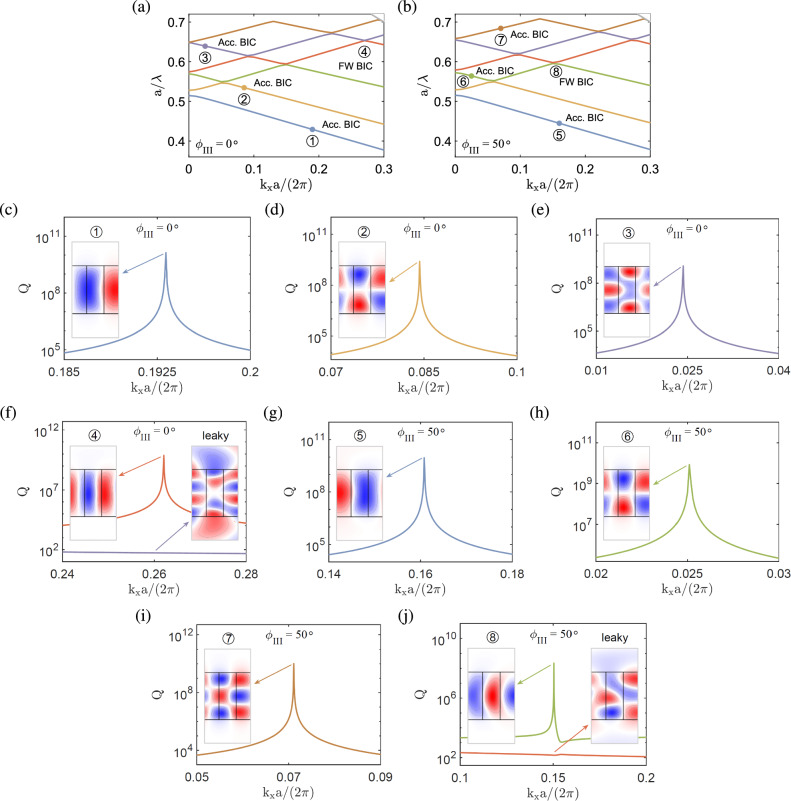
BICs in the anisotropic photonic crystal slab at ${k_x\ne 0}$ in configuration III, with the same geometry material parameters as in Fig. 2. (**a**) and (**d**) Band structures for ${\phi _{\mathrm{{III}}}=0^{\circ }}$ and $50^{\circ }$, respectively. (**c**–**f**) Quality factors and transverse magnetic fields ($\textrm{Re}[H_z]$) of the accidental BICs at $k_xa/\left( 2\pi \right) =0.194, 0.085, 0.0251$ and the FW BIC at $k_xa/\left( 2\pi \right) =0.262$ for ${\phi _{\mathrm{{III}}}=0^{\circ }}$. (**g**–**j**) Quality factors and transverse magnetic fields of the accidental FW BICs at $k_xa/\left( 2\pi \right) =0.161, 0.0251,0.0711$ and the FW BIC at $k_xa/\left( 2\pi \right) =0.15$, respectively, for ${\phi _{\mathrm{{III}}}=50^{\circ }}$.

The band structure of the anisotropic photonic crystal slab at a particular rotation angle ($\phi _{\textrm{I}}=57^\circ$) is shown in Fig. 5d. The avoided crossing occurs between two upper bands (orange and purple lines) at a particular wave number [$k_xa/\left( 2\pi \right) =0.107$], which is labeled by $\textcircled {4}$. Similar features of the FW BIC are shown in Fig. 5f, in which a very large quality factor is attained at its peak on one of the two bands (orange line), and the transverse magnetic fields ($\textrm{Re}[H_z]$) near the eigenmode at $\textcircled {4}$ display a bound state with a quasi-symmetric (TM$_{32}$-like) pattern and a leaky state with a much less concentrated field distribution [cf. insets in Fig. 5f].

**Configuration II.** Figure 6a and b show the band structures of the anisotropic photonic crystal slab in configuration II for zero and a particular rotation angle ($\phi _{\textrm{II}}=0^\circ$ and $45^\circ$), respectively. In Fig. 6c and d, very large quality factors are attained at their peaks on two isolated bands (blue and purple lines) at particular wave numbers [$k_xa/\left( 2\pi \right) =0.0501, 0.138$] for $\phi _{\textrm II}=0^\circ$, which are labeled by $\textcircled {1}$ and $\textcircled {2}$, respectively. The two eigenmodes have the characteristics of accidental BICs similar to those in configuration I. The transverse magnetic fields ($\textrm{Re}[H_z]$) of the eigenmodes at $\textcircled {1}$ and $\textcircled {2}$ display quasi-antisymmetric (TM$_{21}$-like and TM$_{22}$-like) patterns [cf. insets in Fig. 6c and d].

In Fig. 6e and f, very large quality factors are attained at their peaks on two isolated bands (blue and orange lines) at particular wave numbers [$k_xa/\left( 2\pi \right) =0.087, 0.088$] for $\phi _{\textrm{II}}=45^\circ$, which are labeled by $\textcircled {3}$ and $\textcircled {4}$, respectively. The two eigenmodes also have the characteristics of accidental BICs. The transverse magnetic fields ($\textrm{Re}[H_z]$) of the eigenmodes at $\textcircled {3}$ and $\textcircled {4}$ display quasi-antisymmetric (TM$_{21}$-like and TM$_{22}$-like) patterns [cf. insets in Fig. 6e and f]. On the other hand, the avoided crossings occur between two pairs of two interacting bands (green and orange lines, purple and brown lines) at particular wave numbers [$k_xa/\left( 2\pi \right) =0.175, 0.244$], which are labeled by $\textcircled {5}$ and $\textcircled {6}$, respectively. In Fig. 6 g and h, very large quality factors are attained at their peaks near the avoided crossing points, having the characteristics of FW BICs. The transverse magnetic fields ($\textrm{Re}[H_z]$) of the eigenmodes near $\textcircled {5}$ display a bound state with a quasi-antisymmetric (TM$_{21}$-like) pattern and a leaky state, while the transverse magnetic fields for the eigenmodes near $\textcircled {6}$ display a bound state with a quasi-symmetric (TM$_{32}$-like) pattern and a leaky state [cf. insets in Fig. 6g and h].

**Configuration III.** Figure 7a and b show the band structures of the anisotropic photonic crystal slab in configuration III for zero and a particular rotation angle ($\phi _{\textrm{III}}=0^\circ$ and $50^\circ$), respectively. In Fig. 7c–e, very large quality factors are attained at their peaks on three isolated bands (blue, yellow, purple lines) at particular wave numbers [$k_xa/\left( 2\pi \right) =0.194, 0.085, 0.0251$] for $\phi _{\textrm{III}}=0^\circ$, which are labeled by $\textcircled {1}$, $\textcircled {2}$, $\textcircled {3}$, respectively. The three eigenmodes have the characteristics of accidental BICs similar to those in configurations I and II. The transverse magnetic fields ($\textrm{Re}[H_z]$) of the eigenmodes at $\textcircled {1}$, $\textcircled {2}$, $\textcircled {3}$ display quasi-antisymmetric (TM$_{21}$-like, TM$_{22}$-like, TM$_{23}$-like) patterns [cf. insets in Fig. 7c–e]. Note that the symmetry in higher order modes is less obvious, especially in the case of $k_x\ne 0$. On the other hand, the avoided crossing occurs between two upper bands (orange and purple lines) at a particular wave number [$k_xa/\left( 2\pi \right) =0.262$], which are labeled by $\textcircled {4}$. In Fig. 7f, a very large quality factor is attained at its peak near the avoided crossing point, having the characteristics of FW BICs similar to those in configurations I and II. The transverse magnetic fields ($\textrm{Re}[H_z]$) of the eigenmodes near $\textcircled {4}$ display a bound state with a quasi-antisymmetric (TM$_{21}$-like) pattern and a leaky state [cf. insets in Fig. 7f]. Note also that the symmetry pattern is less obvious for $k_x\ne 0$.

In Fig. 7g–i, very large quality factors are attained at their peaks on three isolated bands (blue, green, brown lines) at particular wave numbers [$k_xa/\left( 2\pi \right) =0.161, 0.0251,0.0711$] for $\phi _{\textrm III}=50^\circ$, which are labeled by $\textcircled {5}$, $\textcircled {6}$, $\textcircled {7}$, respectively. The three eigenmodes have the characteristics of accidental BICs similar to those in configurations I and II. The transverse magnetic fields ($\textrm{Re}[H_z]$) of the eigenmodes at $\textcircled {5}$, $\textcircled {6}$, $\textcircled {7}$ display quasi-antisymmetric (TM$_{21}$-like, TM$_{22}$-like, TM$_{23}$-like) patterns [cf. insets in Fig. 7g–i]. On the other hand, the avoided crossing occurs between two upper bands (green and orange lines) at a particular wave number [$k_xa/\left( 2\pi \right) =0.15$], which is labeled by $\textcircled {8}$. In Fig. 7j, a very large quality factor is attained at its peak near the avoided crossing point, having the characteristics of FW BIC similar to those in configurations I and II. The transverse magnetic fields ($\textrm{Re}[H_z]$) of the eigenmode near $\textcircled {8}$ display a bound state with a quasi-symmetric (TM$_{31}$-like) pattern and a leaky state [cf. insets in Fig. 7j].

**Table 1:** Summary of BICs in asymmetric photonic crystal slabs.

Configuration	Optical axis plane	BIC type (${k_x=0}$)	BIC type (${k_x\ne 0}$)
I	*xy* plane	SP (${\phi _{\mathrm{{I}}}}=0^\circ ,90^\circ$) Accidental (${\phi _{\mathrm{{I}}}}\ne 0^\circ$)	Accidental (${\phi _{\mathrm{{I}}}= 0^\circ }$) FW
II	*yz* plane	FW (${\phi _{\mathrm{{II}}}}\ne 0^\circ$)	Accidental FW (${\phi _{\mathrm{{II}}}\ne 0^\circ }$)
III	*xz* plane	Accidental (${\phi _{\mathrm{{III}}}}\ne 0^\circ$)	Accidental FW

Based on the results reported in the above two subsections, various types of BICs in the anisotropic photonic crystal slabs in the present study are summarized in Table 1 for $k_x=0$ and $k_x\ne 0$ in three different configurations. Here, ${\phi _{\mathrm{{N}}}}\ne 0^\circ$ $(\mathrm{N=I,II,III})$ refers to a particular angle of $\phi _{\mathrm{{N}}}$ not equal to zero degree. In the present study, the same height ratio $h/a=1.66$ (see caption in Fig. 2) is used for different configurations, which is the smallest value that supports the occurrence of BICs for all three orientations of the optical axes for the anisotropic material. Here, the ordinary and extraordinary dielectric constants of the anisotropic material do not correspond to real materials, for convenience of analyzing different types of BICs. They are, however, close to those used in Refs.^45,46,49^. Finally, it is worthy of noting that some of the BICs demonstrated by numerical results without the support of theoretical models could be quasi-BICs even with extremely large quality factors. Nevertheless, it is not uncommon to use the terminology of BICs (might not be strictly true BICs) when the quality factors are very large^39,56,59,62^.

In conclusion, we have investigated the BICs in photonic crystal slabs composed of alternating anisotropic and isotropic dielectric materials. Three difference configurations according to the orientation of optical axis plane are proposed for analyzing the BICs based on the dispersion bands and transmittance diagram. Various types of BICs with very large quality factors and vanishing spectral linewidths are identified in the underlying structure. In particular, SP BICs associated with antisymmetric field patterns occur for zero rotation angle at the Brillouin zone center. Accidental BICs emerge on isolated bands while FW BICs appear near the avoided crossing between two interacting bands for zero or particular rotation angles at or off the Brillouin zone center.

## Data Availability

All data generated or analysed during this study are included in this published article.
